# The Effect of Lithium on the Structure and Function of the Human Retina: A Systematic Review

**DOI:** 10.1192/bjo.2026.11171

**Published:** 2026-06-30

**Authors:** Nicole Needham, David Grossett, Jasna Martinovic, Thomas MacGillivray, Daniel Smith

**Affiliations:** 1 University of Edinburgh, Edinburgh, United Kingdom; 2 NHS Lothian, Edinburgh, United Kingdom

## Abstract

**Aims:**

Lithium is the gold standard treatment for bipolar disorder, but its mechanism of action is not fully understood. Emerging research suggests structural and functional alterations in the retina of people with bipolar disorder and depression, including a thinner retinal nerve fibre layer and ganglion cell layer. Retinal structure and function can be characterised using several noninvasive imaging techniques, from which numerous quantitative metrics can be derived. We aimed to complete a systematic review of the literature to establish which parameters have been evaluated in relation to lithium use and what insights they provide into its potential retinal effects.

**Methods:**

Searches using the terms ‘Lithium’ AND ‘retina’ were carried out to identify peer reviewed studies assessing the impact of lithium on retinal structure or function. These included those with or without a control group comparison, pre- and post-lithium comparisons and observational studies. There were no exclusions based on the quantity or preparation of lithium administered, or the length of administration. Any studies where lithium was administered in combination with another intervention were excluded, unless it was directly compared to the other intervention without lithium. The main outcome of interest was optical coherence tomography (OCT) derived metrics, but any measure of structure or function was included.

**Results:**

Seven studies reporting structural outcomes, and 10 reporting functional outcomes, were identified.Structural outcomes were derived from optical coherence tomography (OCT) imaging studies which had multiple limitations. There was no evidence of statistically significant difference in the retinal nerve fibre layer (RNFL) between people with bipolar disorder taking lithium and healthy controls in two studies with larger numbers, indicating a protective effect on the retina. In four studies looking at differences in people with bipolar taking lithium and those taking valproate, two showed no signs of difference and two evidence of statistically thicker RNFL in the lithium group, also in line with a potential protective effect. For all other OCT derived metrics, the evidence was limited.

Studies reporting functional outcomes were highly heterogeneous with multiple limitations and no clear trends. The majority reported a statistically significant effect of lithium on at least one functional measure, derived from electrooculography, electroretinography, and dark adaptation threshold procedures.

**Conclusion:**

Trends indicate that lithium is likely to have an effect on both the structure and function of the retina, but significant limitations in all included studies mean that better designed and adequately powered prospective studies are required for both structural and functional measures of the retina.

